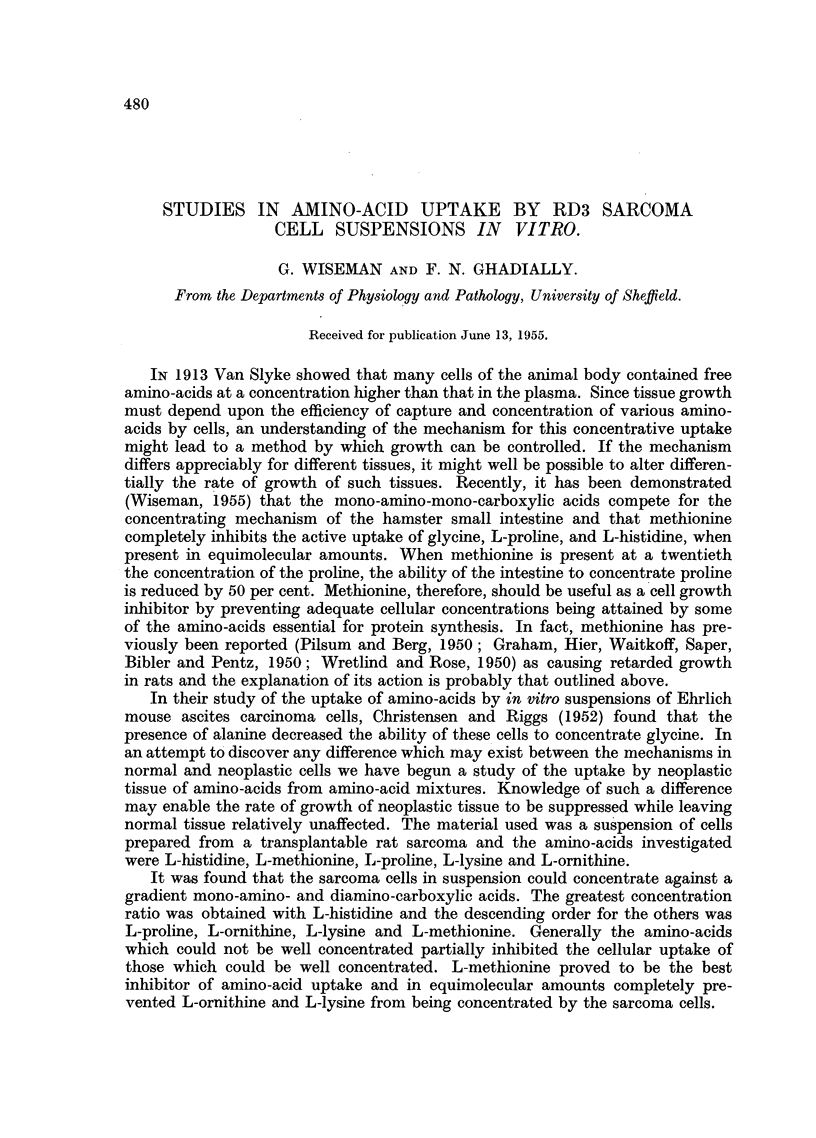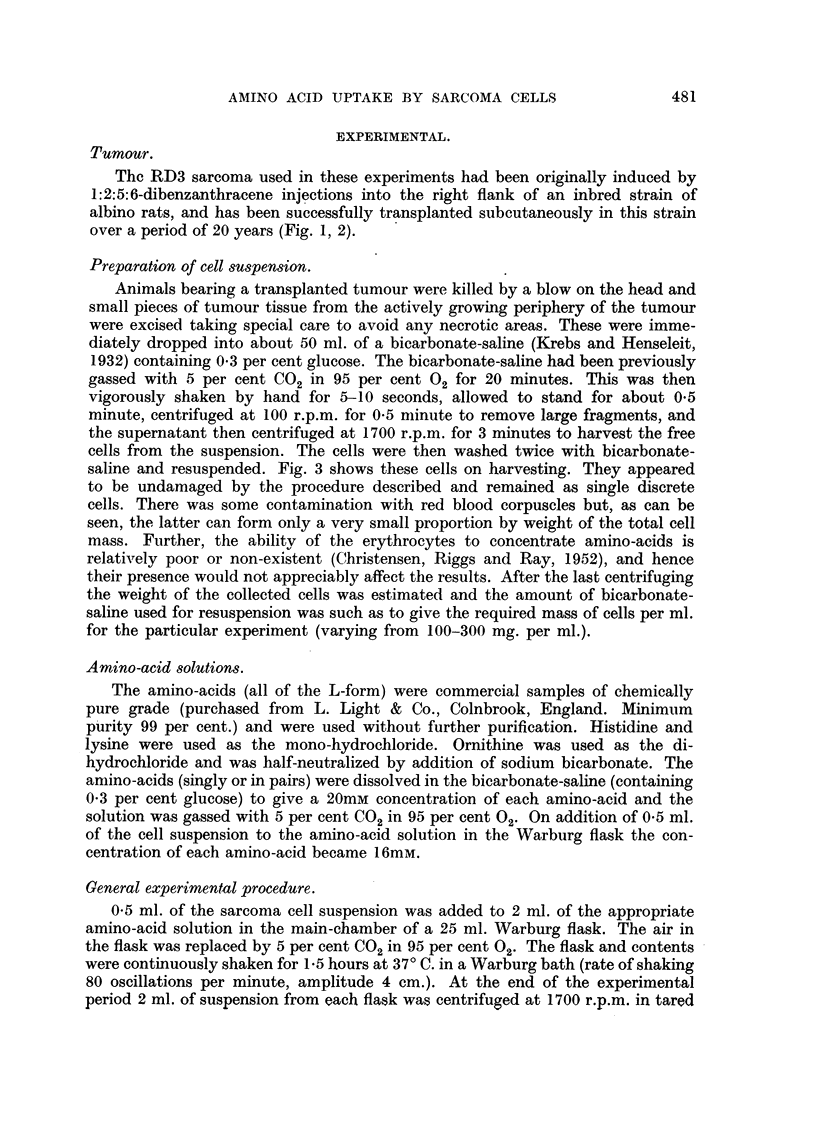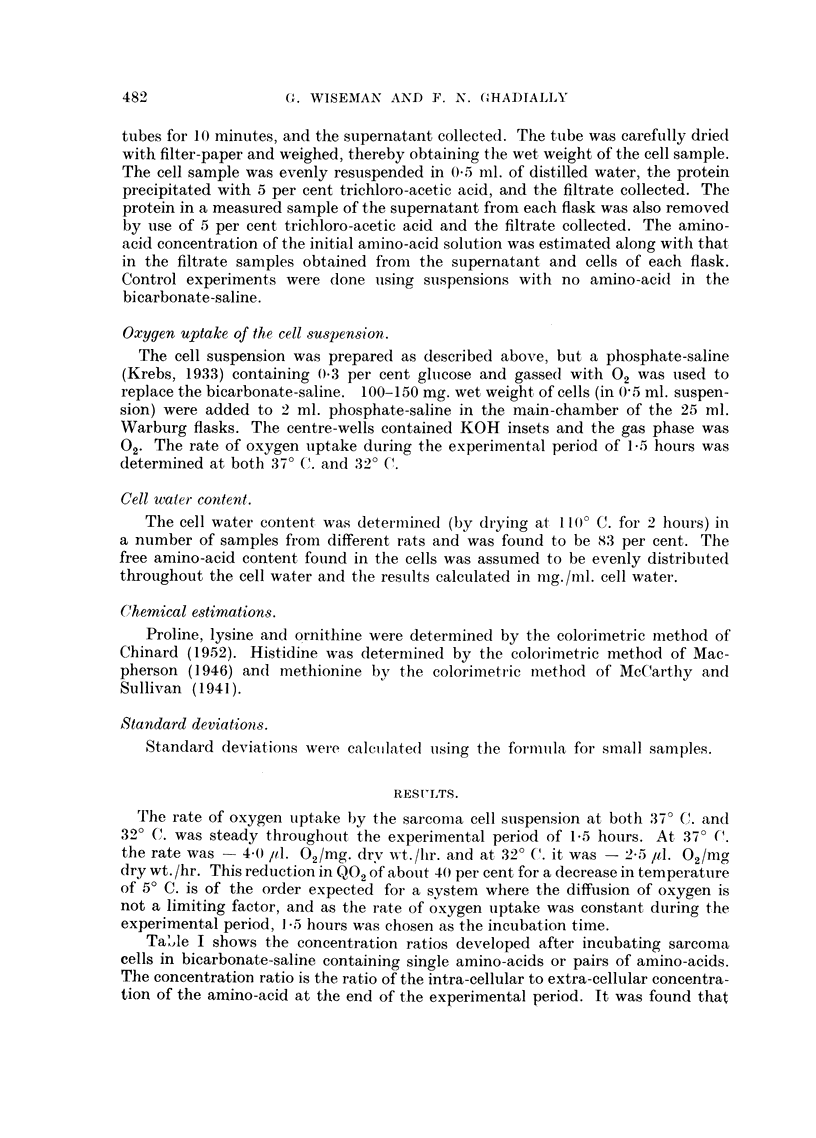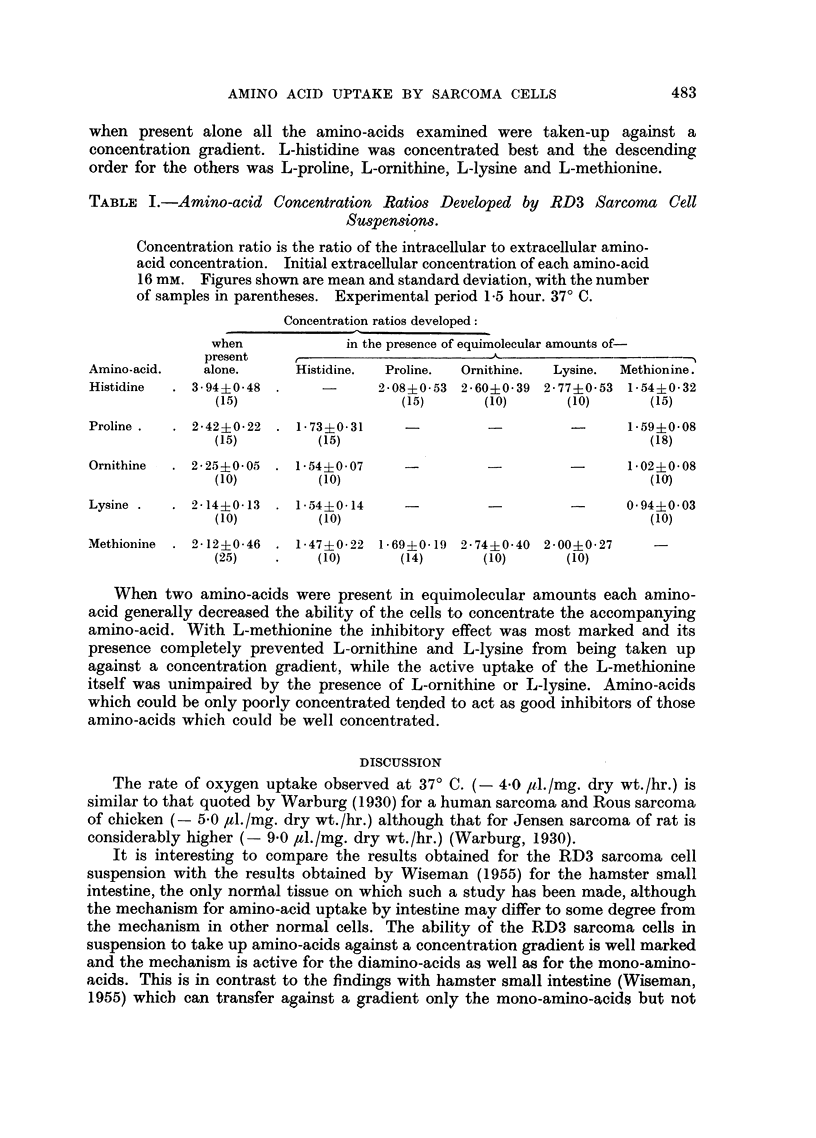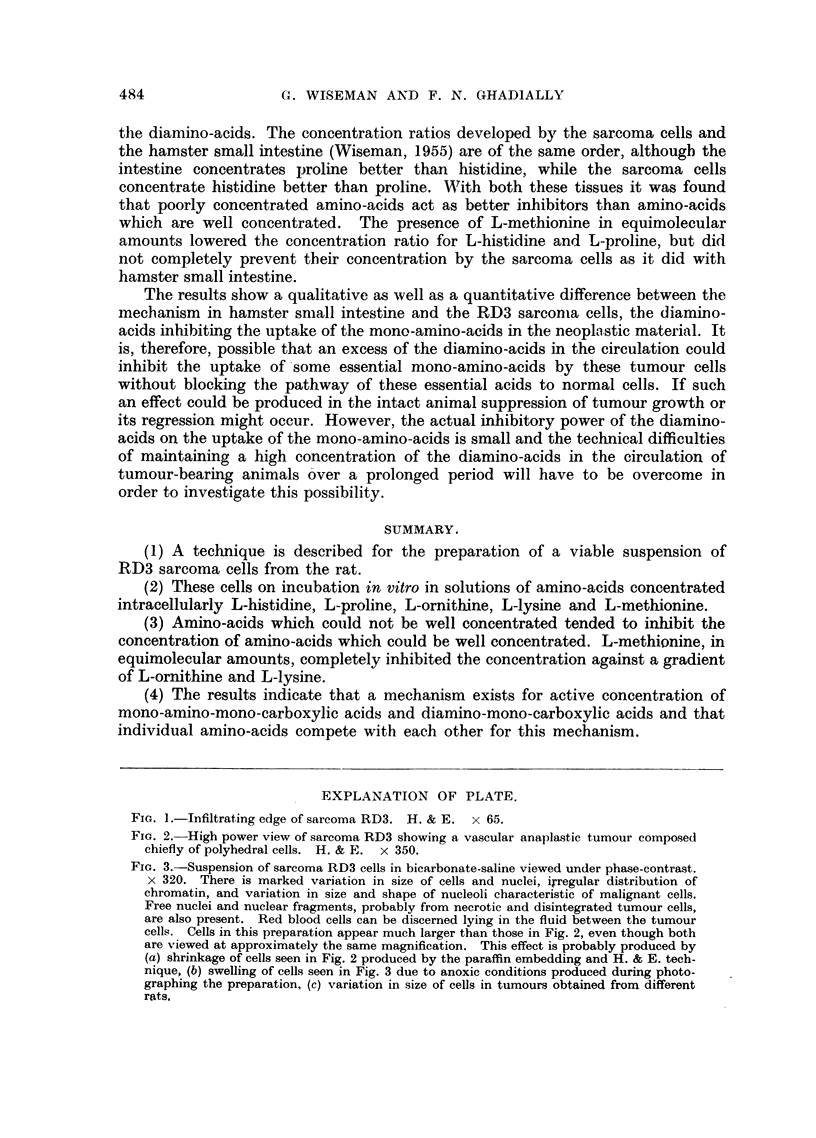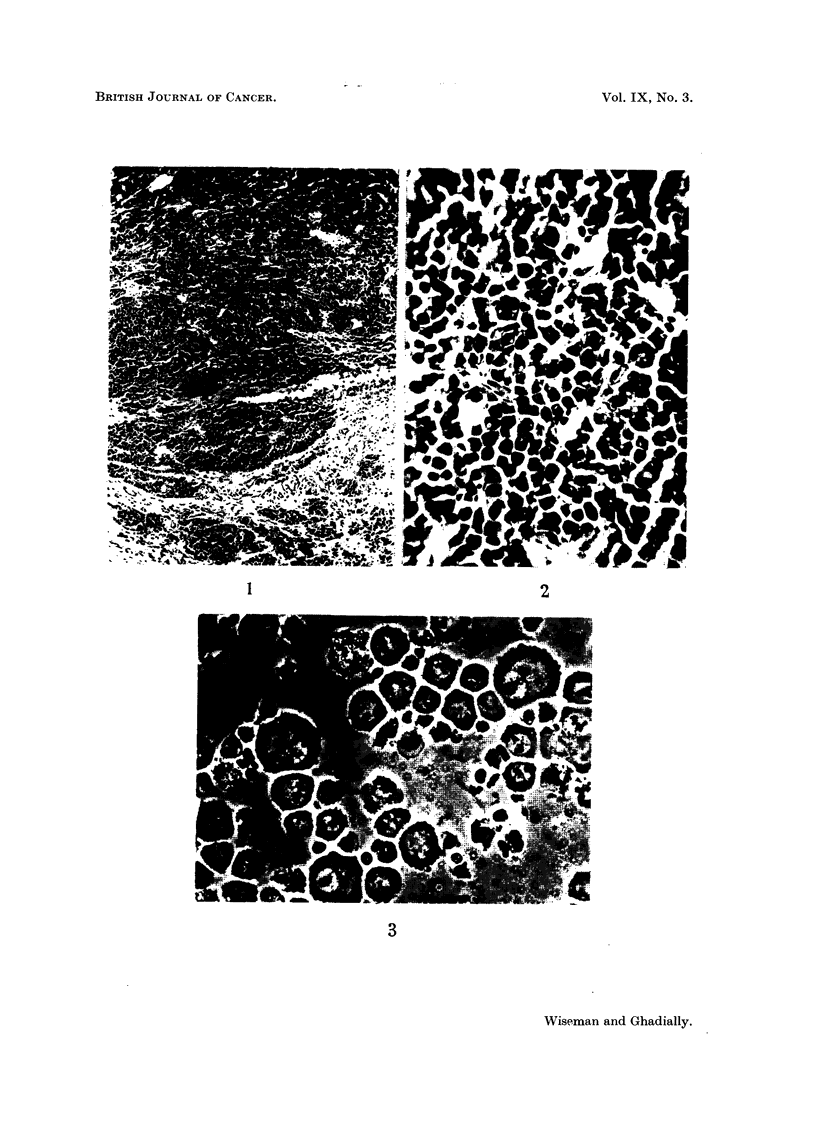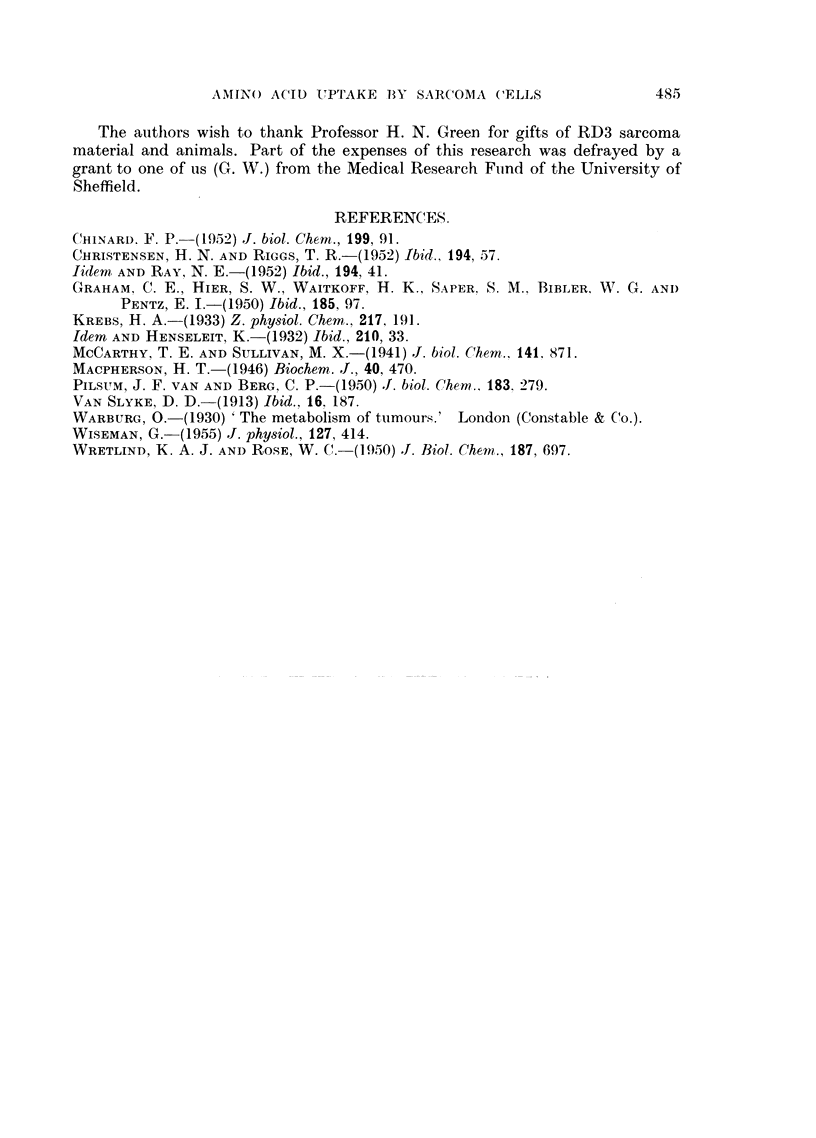# Studies in Amino-acid Uptake by RD3 Sarcoma Cell Suspensions In Vitro

**DOI:** 10.1038/bjc.1955.50

**Published:** 1955-09

**Authors:** G. Wiseman, F. N. Ghadially

## Abstract

**Images:**


### STUDIES IN AMINO-ACID UPTAKE BY RD3 SARCOMA CELL SUSPENSIONS *IN VITRO.*

G. WISEMAN AND F. N. GHADIALLY.

*From the Departments of Physiology and Pathology, University of Sheffield.*

Received for publication June 13, 1955.

IN 1913 Van Slyke showed that many cells of the animal body contained free amino-acids at a concentration higher than that in the plasma. Since tissue growth must depend upon the efficiency of capture and concentration of various amino-acids by cells, an understanding of the mechanism for this concentrative uptake might lead to a method by which growth can be controlled. If the mechanism differs appreciably for different tissues, it might well be possible to alter differentially the rate of growth of such tissues. Recently, it has been demonstrated (Wiseman, 1955) that the mono-amino-mono-carboxylic acids compete for the concentrating mechanism of the hamster small intestine and that methionine completely inhibits the active uptake of glycine, L-proline, and L-histidine, when present in equimolecular amounts. When methionine is present at a twentieth the concentration of the proline, the ability of the intestine to concentrate proline is reduced by 50 per cent. Methionine, therefore, should be useful as a cell growth inhibitor by preventing adequate cellular concentrations being attained by some of the amino-acids essential for protein synthesis. In fact, methionine has previously been reported (Pilsum and Berg, 1950 ; Graham, Hier, Waitkoff, Saper, Bibler and Pentz, 1950 ; Wretlind and Rose, 1950) as causing retarded growth in rats and the explanation of its action is probably that outlined above.

In their study of the uptake of amino-acids by *in vitro* suspensions of Ehrlich mouse ascites carcinoma cells, Christensen and Riggs (1952) found that the presence of alanine decreased the ability of these cells to concentrate glycine. In an attempt to discover any difference which may exist between the mechanisms in normal and neoplastic cells we have begun a study of the uptake by neoplastic tissue of amino-acids from amino-acid mixtures. Knowledge of such a difference may enable the rate of growth of neoplastic tissue to be suppressed while leaving normal tissue relatively unaffected. The material used was a suspension of cells prepared from a transplantable rat sarcoma and the amino-acids investigated were L-histidine, L-methionine, L-proline, L-lysine and L-ornithine.

It was found that the sarcoma cells in suspension could concentrate against a gradient mono-amino- and diamino-carboxylic acids. The greatest concentration ratio was obtained with L-histidine and the descending order for the others was L-proline, L-ornithine, L-lysine and L-methionine. Generally the amino-acids which could not be well concentrated partially inhibited the cellular uptake of those which could be well concentrated. L-methionine proved to be the best inhibitor of amino-acid uptake and in equimolecular amounts completely prevented L-ornithine and L-lysine from being concentrated by the sarcoma cells.

### EXPERIMENTAL.

*Tumour.*

Thc RD3 sarcoma used in these experiments had been originally induced by 1:2:5:6-dibenzanthracene injections into the right flank of an inbred strain of albino rats, and has been successfully transplanted subcutaneously in this strain over a period of 20 years (Fig. 1, 2).

*Preparation of cell suspension.*

Animals bearing a transplanted tumour were killed by a blow on the head and small pieces of tumour tissue from the actively growing periphery of the tumour were excised taking special care to avoid any necrotic areas. These were immediately dropped into about 50 ml. of a bicarbonate-saline (Krebs and Henseleit, 1932) containing 0·3 per cent glucose. The bicarbonate-saline had been previously gassed with 5 per cent $CO_2$ in 95 per cent $O_2$ for 20 minutes. This was then vigorously shaken by hand for 5–10 seconds, allowed to stand for about 0·5 minute, centrifuged at 100 r.p.m. for 0·5 minute to remove large fragments, and the supernatant then centrifuged at 1700 r.p.m. for 3 minutes to harvest the free cells from the suspension. The cells were then washed twice with bicarbonate-saline and resuspended. Fig. 3 shows these cells on harvesting. They appeared to be undamaged by the procedure described and remained as single discrete cells. There was some contamination with red blood corpuscles but, as can be seen, the latter can form only a very small proportion by weight of the total cell mass. Further, the ability of the erythrocytes to concentrate amino-acids is relatively poor or non-existent (Christensen, Riggs and Ray, 1952), and hence their presence would not appreciably affect the results. After the last centrifuging the weight of the collected cells was estimated and the amount of bicarbonate-saline used for resuspension was such as to give the required mass of cells per ml. for the particular experiment (varying from 100–300 mg. per ml.).

*Amino-acid solutions.*

The amino-acids (all of the L-form) were commercial samples of chemically pure grade (purchased from L. Light & Co., Colnbrook, England. Minimum purity 99 per cent.) and were used without further purification. Histidine and lysine were used as the mono-hydrochloride. Ornithine was used as the di-hydrochloride and was half-neutralized by addition of sodium bicarbonate. The amino-acids (singly or in pairs) were dissolved in the bicarbonate-saline (containing 0·3 per cent glucose) to give a 20mM concentration of each amino-acid and the solution was gassed with 5 per cent $CO_2$ in 95 per cent $O_2$. On addition of 0·5 ml. of the cell suspension to the amino-acid solution in the Warburg flask the concentration of each amino-acid became 16mM.

*General experimental procedure.*

0·5 ml. of the sarcoma cell suspension was added to 2 ml. of the appropriate amino-acid solution in the main-chamber of a 25 ml. Warburg flask. The air in the flask was replaced by 5 per cent $CO_2$ in 95 per cent $O_2$. The flask and contents were continuously shaken for 1·5 hours at 37° C. in a Warburg bath (rate of shaking 80 oscillations per minute, amplitude 4 cm.). At the end of the experimental period 2 ml. of suspension from each flask was centrifuged at 1700 r.p.m. in tared tubes for 10 minutes, and the supernatant collected. The tube was carefully dried with filter-paper and weighed, thereby obtaining the wet weight of the cell sample. The cell sample was evenly resuspended in 0·5 ml. of distilled water, the protein precipitated with 5 per cent trichloro-acetic acid, and the filtrate collected. The protein in a measured sample of the supernatant from each flask was also removed by use of 5 per cent trichloro-acetic acid and the filtrate collected. The amino-acid concentration of the initial amino-acid solution was estimated along with that in the filtrate samples obtained from the supernatant and cells of each flask. Control experiments were done using suspensions with no amino-acid in the bicarbonate-saline.

*Oxygen uptake of the cell suspension.*

The cell suspension was prepared as described above, but a phosphate-saline (Krebs, 1933) containing 0·3 per cent glucose and gassed with $O_2$ was used to replace the bicarbonate-saline. 100–150 mg. wet weight of cells (in 0·5 ml. suspension) were added to 2 ml. phosphate-saline in the main-chamber of the 25 ml. Warburg flasks. The centre-wells contained KOH insets and the gas phase was $O_2$. The rate of oxygen uptake during the experimental period of 1·5 hours was determined at both 37° C. and 32° C.

*Cell water content.*

The cell water content was determined (by drying at 110° C. for 2 hours) in a number of samples from different rats and was found to be 83 per cent. The free amino-acid content found in the cells was assumed to be evenly distributed throughout the cell water and the results calculated in mg./ml. cell water.

*Chemical estimations.*

Proline, lysine and ornithine were determined by the colorimetric method of Chinard (1952). Histidine was determined by the colorimetric method of Macpherson (1946) and methionine by the colorimetric method of McCarthy and Sullivan (1941).

*Standard deviations.*

Standard deviations were calculated using the formula for small samples.

### RESULTS.

The rate of oxygen uptake by the sarcoma cell suspension at both 37° C. and 32° C. was steady throughout the experimental period of 1·5 hours. At 37° C. the rate was — 4·0 $\mu$l. $O_2$/mg. dry wt./hr. and at 32° C. it was — 2·5 $\mu$l. $O_2$/mg dry wt./hr. This reduction in $QO_2$ of about 40 per cent for a decrease in temperature of 5° C. is of the order expected for a system where the diffusion of oxygen is not a limiting factor, and as the rate of oxygen uptake was constant during the experimental period, 1·5 hours was chosen as the incubation time.

Table I shows the concentration ratios developed after incubating sarcoma cells in bicarbonate-saline containing single amino-acids or pairs of amino-acids. The concentration ratio is the ratio of the intra-cellular to extra-cellular concentration of the amino-acid at the end of the experimental period. It was found that when present alone all the amino-acids examined were taken-up against a concentration gradient. L-histidine was concentrated best and the descending order for the others was L-proline, L-ornithine, L-lysine and L-methionine.

TABLE I.—*Amino-acid Concentration Ratios Developed by RD3 Sarcoma Cell Suspensions.*

Concentration ratio is the ratio of the intracellular to extracellular amino-acid concentration. Initial extracellular concentration of each amino-acid 16 mM. Figures shown are mean and standard deviation, with the number of samples in parentheses. Experimental period 1·5 hour. 37° C.

| | Concentration ratios developed: | | | | | |
|---|---|---|---|---|---|---|
| | when present alone | in the presence of equimolecular amounts of— | | | | |
| Amino-acid. | | Histidine. | Proline. | Ornithine. | Lysine. | Methionine. |
| Histidine | 3·94±0·48 (15) | — | 2·08±0·53 (15) | 2·60±0·39 (10) | 2·77±0·53 (10) | 1·54±0·32 (15) |
| Proline | 2·42±0·22 (15) | 1·73±0·31 (15) | — | — | — | 1·59±0·08 (18) |
| Ornithine | 2·25±0·05 (10) | 1·54±0·07 (10) | — | — | — | 1·02±0·08 (10) |
| Lysine | 2·14±0·13 (10) | 1·54±0·14 (10) | — | — | — | 0·94±0·03 (10) |
| Methionine | 2·12±0·46 (25) | 1·47±0·22 (10) | 1·69±0·19 (14) | 2·74±0·40 (10) | 2·00±0·27 (10) | — |

When two amino-acids were present in equimolecular amounts each amino-acid generally decreased the ability of the cells to concentrate the accompanying amino-acid. With L-methionine the inhibitory effect was most marked and its presence completely prevented L-ornithine and L-lysine from being taken up against a concentration gradient, while the active uptake of the L-methionine itself was unimpaired by the presence of L-ornithine or L-lysine. Amino-acids which could be only poorly concentrated tended to act as good inhibitors of those amino-acids which could be well concentrated.

### DISCUSSION

The rate of oxygen uptake observed at 37° C. (— 4·0 μl./mg. dry wt./hr.) is similar to that quoted by Warburg (1930) for a human sarcoma and Rous sarcoma of chicken (— 5·0 μl./mg. dry wt./hr.) although that for Jensen sarcoma of rat is considerably higher (— 9·0 μl./mg. dry wt./hr.) (Warburg, 1930).

It is interesting to compare the results obtained for the RD3 sarcoma cell suspension with the results obtained by Wiseman (1955) for the hamster small intestine, the only normal tissue on which such a study has been made, although the mechanism for amino-acid uptake by intestine may differ to some degree from the mechanism in other normal cells. The ability of the RD3 sarcoma cells in suspension to take up amino-acids against a concentration gradient is well marked and the mechanism is active for the diamino-acids as well as for the mono-amino-acids. This is in contrast to the findings with hamster small intestine (Wiseman, 1955) which can transfer against a gradient only the mono-amino-acids but not the diamino-acids. The concentration ratios developed by the sarcoma cells and the hamster small intestine (Wiseman, 1955) are of the same order, although the intestine concentrates proline better than histidine, while the sarcoma cells concentrate histidine better than proline. With both these tissues it was found that poorly concentrated amino-acids act as better inhibitors than amino-acids which are well concentrated. The presence of L-methionine in equimolecular amounts lowered the concentration ratio for L-histidine and L-proline, but did not completely prevent their concentration by the sarcoma cells as it did with hamster small intestine.

The results show a qualitative as well as a quantitative difference between the mechanism in hamster small intestine and the RD3 sarcoma cells, the diamino-acids inhibiting the uptake of the mono-amino-acids in the neoplastic material. It is, therefore, possible that an excess of the diamino-acids in the circulation could inhibit the uptake of some essential mono-amino-acids by these tumour cells without blocking the pathway of these essential acids to normal cells. If such an effect could be produced in the intact animal suppression of tumour growth or its regression might occur. However, the actual inhibitory power of the diamino-acids on the uptake of the mono-amino-acids is small and the technical difficulties of maintaining a high concentration of the diamino-acids in the circulation of tumour-bearing animals over a prolonged period will have to be overcome in order to investigate this possibility.

### SUMMARY.

(1) A technique is described for the preparation of a viable suspension of RD3 sarcoma cells from the rat.

(2) These cells on incubation *in vitro* in solutions of amino-acids concentrated intracellularly L-histidine, L-proline, L-ornithine, L-lysine and L-methionine.

(3) Amino-acids which could not be well concentrated tended to inhibit the concentration of amino-acids which could be well concentrated. L-methionine, in equimolecular amounts, completely inhibited the concentration against a gradient of L-ornithine and L-lysine.

(4) The results indicate that a mechanism exists for active concentration of mono-amino-mono-carboxylic acids and diamino-mono-carboxylic acids and that individual amino-acids compete with each other for this mechanism.

---

### EXPLANATION OF PLATE.

FIG. 1.—Infiltrating edge of sarcoma RD3. H. & E. × 65.

FIG. 2.—High power view of sarcoma RD3 showing a vascular anaplastic tumour composed chiefly of polyhedral cells. H. & E. × 350.

FIG. 3.—Suspension of sarcoma RD3 cells in bicarbonate-saline viewed under phase-contrast. × 320. There is marked variation in size of cells and nuclei, irregular distribution of chromatin, and variation in size and shape of nucleoli characteristic of malignant cells. Free nuclei and nuclear fragments, probably from necrotic and disintegrated tumour cells, are also present. Red blood cells can be discerned lying in the fluid between the tumour cells. Cells in this preparation appear much larger than those in Fig. 2, even though both are viewed at approximately the same magnification. This effect is probably produced by (*a*) shrinkage of cells seen in Fig. 2 produced by the paraffin embedding and H. & E. technique, (*b*) swelling of cells seen in Fig. 3 due to anoxic conditions produced during photographing the preparation, (*c*) variation in size of cells in tumours obtained from different rats.

1

2

3

Wiseman and Ghadially.

The authors wish to thank Professor H. N. Green for gifts of RD3 sarcoma material and animals. Part of the expenses of this research was defrayed by a grant to one of us (G. W.) from the Medical Research Fund of the University of Sheffield.

### REFERENCES.

CHINARD, F. P.—(1952) *J. biol. Chem.*, **199**, 91.
CHRISTENSEN, H. N. AND RIGGS, T. R.—(1952) *Ibid.*, **194**, 57.
*Iidem* AND RAY, N. E.—(1952) *Ibid.*, **194**, 41.
GRAHAM, C. E., HIER, S. W., WAITKOFF, H. K., SAPER, S. M., BIBLER, W. G. AND PENTZ, E. I.—(1950) *Ibid.*, **185**, 97.
KREBS, H. A.—(1933) *Z. physiol. Chem.*, **217**, 191.
*Idem* AND HENSELEIT, K.—(1932) *Ibid.*, **210**, 33.
McCARTHY, T. E. AND SULLIVAN, M. X.—(1941) *J. biol. Chem.*, **141**, 871.
MACPHERSON, H. T.—(1946) *Biochem. J.*, **40**, 470.
PILSUM, J. F. VAN AND BERG, C. P.—(1950) *J. biol. Chem.*, **183**, 279.
VAN SLYKE, D. D.—(1913) *Ibid.*, **16**, 187.
WARBURG, O.—(1930) 'The metabolism of tumours.' London (Constable & Co.).
WISEMAN, G.—(1955) *J. physiol.*, **127**, 414.
WRETLIND, K. A. J. AND ROSE, W. C.—(1950) *J. Biol. Chem.*, **187**, 697.